# Quantification of microRNA-210 in the cerebrospinal fluid and serum: Implications for Alzheimer’s disease

**DOI:** 10.3892/etm.2015.2179

**Published:** 2015-01-14

**Authors:** YAN ZHU, CHENGSHAN LI, AICHEN SUN, YUANYE WANG, SHENGNIAN ZHOU

**Affiliations:** 1 Department of ICU, Zaozhuang Municipal Hospital, Zaozhuang, Shandong 277102, P.R. China; 2 Department of Neurology, Qilu Hospital of Shandong University, Jinan, Shandong 250012, P.R. China

**Keywords:** Alzheimer’s disease, microRNA-210, vascular endothelial growth factor, genetic marker

## Abstract

The aim of the present study was to investigate the potential clinical application of the genetic marker microRNA (miRNA)-210 in the cerebrospinal fluid (CSF) and serum of patients with Alzheimer’s disease (AD). The enrolled patients were divided into the mild cognitive impairment (MCI) and AD groups. Healthy individuals were used as the controls. The mRNA and protein expression of vascular endothelial growth factor (VEGF) in the CSF and serum samples was detected by reverse transcription-quantitative polymerase chain reaction (RT-qPCR) and western blot analysis, respectively. The expression of miRNA-210 in the CSF and serum was detected by RT-qPCR. The results revealed that the mRNA and protein expression levels of VEGF in the CSF and serum were decreased in the MCI and AD groups compared with those in the control group. The greater the severity of the dementia, the lower the mRNA and protein expression of VEGF. Similar to the trend observed for VEGF, the miRNA-210 expression in the CSF and serum decreased as the severity of the AD increased. miRNA-210 is thus not only indicative of AD pathogenesis, but may also provide novel insights into the prevention and treatment of the disease.

## Introduction

Alzheimer’s disease (AD) is a progressive, neurodegenerative disease, which is pathologically characterized by senile plaques, neurofibrillary tangles and neural cell death. The etiology and pathogenesis of the disease remain unknown (1). It is generally believed that cerebral ischemia and hypoxia, caused by head trauma, brain vascular inflammation, cerebral vascular stenosis or partial embolism, contribute to the disease onset and development (2); therefore, the study of brain vascular lesions has always been an important topic in research into AD.

Vascular endothelial growth factor (VEGF) is one of the most important regulatory factors in vascular growth and development. VEGF can promote angiogenesis and increase the blood supply to support metabolic processes (3). It has been found in a previous study that the expression of VEGF is altered in the occurrence and development of AD, which may be associated with the disease process (4). Recently, the role of microRNA (miRNA) in post-transcriptional gene regulation has been uncovered (5). VEGF has been observed to be one of the targets of miRNA-210 (6), and a previous study has shown that the upregulation of miRNA-210 can increase the expression of VEGF in kidney tissue (7). Due to its stability and specificity, miRNA-210 has been used as a genetic marker for the early diagnosis and treatment of VEGF-associated diseases; however, the quantitative detection of miRNA-210 in the cerebrospinal fluid (CSF) and serum in AD, particularly regarding the regulation of VEGF, has, to the best of our knowledge, not been fully elucidated.

In the present study, the expression levels of VEGF and miRNA-210 in the CSF and serum of patients with AD were detected, and their association with the disease severity was investigated.

## Materials and methods

### Patients and sample collection

A total of 56 patients with dementia, who had been admitted to Zaozhuang Municipal Hospital between January 2012 and August 2013, were enrolled in the present study. Based on the National Institute of Neurological and Communicative Disorders and Stroke and the Alzheimer’s Disease and Related Disorders Association (NINCDS-ADRDA) diagnostic criteria amendment, published by the National Institute on Aging and the Alzheimer’s Association (NIA-AA) in April 2011 (8), these patients were diagnosed with AD and categorized into the mild cognitive impairment (MCI) (n=30) and AD (n=26) groups. A total of 42 healthy individuals were used as controls. The MCI group comprised 18 male and 12 female patients, with ages ranging from 61 to 82 years (mean age, 71.6 years); the AD group comprised 12 male and 14 female patients, with ages ranging from 60 to 84 years (mean age, 72.3 years); and the control group comprised 23 male and 19 female subjects, with ages ranging from 62 to 85 years (mean age, 71.9 years). Prior written and informed consent was obtained from every patient prior to their participation in the current study, and the study was approved by the Ethics Review Board of Zaozhuang Municipal Hospital (Zaozhuang, China). Furthermore, eight patients with AD, with sufficient finances, underwent intracranial pressure monitoring, brain oxygen tension monitoring and computed tomography (CT).

For sample collection, 2 ml CSF was extracted from each patient in the morning, centrifuged at 1,200 × g for 5 min at 4°C and then stored at −20°C until required. For the serum samples, peripheral blood was collected from each patient in the morning, after fasting for 12 h. The serum was separated by centrifugation at 12,000 × g for 5 min at 4°C and the samples were stored at −80°C until required.

### Intracranial pressure monitoring

Intracranial pressure was detected by inserting an intraventricular catheter with a sensor (ICP Express; Codman & Shurtleff, Inc., Raynham, MA, USA), into the lateral ventricle of patients with AD. This area of the brain contains liquid that protects the brain and spinal cord. The intracranial pressure was monitored whilst the fluid was drained out through the catheter, which was connected to an Intracranial Pressure Monitoring system (Spiegelberg GmbH & Co. KG, Hamburg, Germany).

### Brain oxygen tension monitoring

Following calibration to anoxia and ambient oxygen, an oval polymethylmethacrylate conformer, containing a conjunctival sensor (LICOX CMP system; Integra Neurosciences, Inc., San Diego, CA, USA), was placed on the superolateral side of the head in order to allow the sensor to engage the superolateral intracranial conjunctiva, while preventing contact between the conformer and the cornea. The sensor was then connected to the conjunctival oxygen monitor (TO2M 2000 Tissue Oxygen Monitoring system; Biomedical Sensors, High Wycombe, UK).

### Computed tomography (CT)

All CT scans were obtained on a single-detector helical HiSpeed CT/i scanner (GE Medical Systems, Milwaukee, WI, USA). All subjects were subjected to a nonenhanced head CT (axial 5-mm collimation), following placement of a brain-tissue oxygen probe. The axial level corresponding to the most inferior aspect of the probe was used. CT perfusion images were acquired by Advantage Workstation (GE Medical Systems).

### Reverse transcription-quantitative polymerase chain reaction (RT-qPCR)

Total RNA in the CSF and serum samples was extracted using TRIzol^®^ (Invitrogen Life Technologies, Carlsbad, CA, USA) with miRcute miRNA isolation kits (Tiangen Biotech, Beijing, China). The purity of the samples was measured at A260/A280 with an ultraviolet spectrophotometer (DR 6000™ UV VIS; HACH, Loveland, CO, USA), and cDNA was obtained by reverse transcription with an miRcute miRNA First-Strand cDNA Synthesis kit (Tiangen Biotech). The RT-qPCR was performed with an iQ5 RT-qPCR detection system (Bio-Rad Laboratories, Inc., Hercules, CA, USA). The reaction conditions for VEGF were as follows: Pre-denaturation at 94°C for 2 min; denaturation at 94°C for 30 sec, annealing at 55°C for 30 sec and extension at 71°C for 1 min, (35 cycles); and a final extension step at 71°C for 2 min. The RT-qPCR reaction conditions for miRNA-210 detection were as follows: Pre-denaturation at 95°C for 10 min; denaturation at 95°C for 15 sec, annealing at 60°C for 1 min and extension at 72°C for 2 min (40 cycles); and a final extension step at 72°C for 7 min. Results were calculated using the 2^−ΔΔCt^ method, and β-actin and U6 were used as the controls for VEGF and miRNA-210, respectively. The RT-qPCR primers (Tiangen Biotech) for the measurement of VEGF mRNA and miRNA-210 expression are listed in Table I.

### Western blot analysis

The total proteins in the CSF and serum samples were extracted with protein lysis solution (Tiangen Biotech). Protein concentration was measured with a bicinchoninic acid kit (Tiangen Biotech). SDS-PAGE sample buffer (Tiangen Biotech) was added to the protein samples, and the samples were boiled for 5 min. A total of 20 μg protein was used for SDS-PAGE with 10% acrylamide gels, and the protein was then transferred onto a membrane at a constant voltage of 100 V for 2 h under ice-cold conditions. The membrane was blocked with 5% skimmed milk for 1 h at room temperature. Rabbit anti-human monoclonal anti-VEGF primary antibody (1:1,000; cat. no. ab52917, Abcam, Cambridge, MA, USA) and the internal reference rabbit anti-human monoclonal anti-β-actin primary antibody (1:5,000; cat. no. ab115777, Abcam) were added for incubation with the membrane at 4°C overnight. A goat anti-rabbit polyclonal immunoglobulin G secondary antibody (1:3,000; cat. no. ab97047, Abcam) was subsequently added for incubation at room temperature for 1 h. The membrane was placed in an enhanced chemiluminescence solution (Tiangen Biotech) and then exposed to the Gel Doc™ EZ Imager (Bio-Rad Laboratories, Inc.). Image Lab software version 3.0 (Bio-Rad Laboratories, Inc.) was used for protein band analysis. The relative content of the target protein was determined as the ratio to the internal reference β-actin.

### Statistical analysis

Data are expressed as the mean ± standard deviation. All data were processed using SPSS 18.0 software (SPSS, Inc., Chicago, IL, USA). One-way analysis of variance was performed for multiple comparisons. P<0.05 was considered to indicate a statistically significant difference.

## Results

### Expression levels of VEGF mRNA and protein are decreased in the CSF of patients with MCI and AD

**Figure 1:**
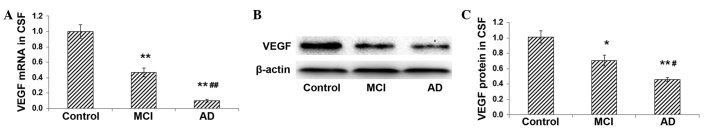
Changes in the expression levels of VEGF mRNA and protein in the CSF of patients with dementia. (A) mRNA expression of VEGF in the CSF was measured by the reverse transcription-quantitative polymerase chain reaction. (B) VEGF protein expression in the CSF of patients with dementia was detected through western blot analysis. (C) Statistical analysis of VEGF protein expression in the CSF. Compared with the control group, ^*^P<0.05 and ^**^P<0.01; compared with the MCI group, ^#^P<0.05 and ^##^P<0.01. MCI, mild cognitive impairment; AD, Alzheimer’s disease; VEGF, vascular endothelial growth factor; CSF, cerebrospinal fluid.

To investigate the role of VEGF in the pathogenesis of AD, the mRNA and protein expression levels of VEGF in the CSF were detected with RT-qPCR and western blot analysis, respectively. The enrolled patients were divided into the MCI (n=30) and AD (n=26) groups, and normal individuals (n=42) were used as controls. Results from the RT-qPCR demonstrated that, compared with the expression in the control group, VEGF mRNA expression in the CSF in the MCI group was significantly reduced (P<0.01), and was further reduced in the AD group (AD versus control, P<0.01; AD versus MCI, P<0.01) (Fig. 1A). Similar results were obtained with the western blot analysis. The protein expression of VEGF was significantly lower in the MCI and AD groups compared with that in the control group (control versus MCI, P<0.05; control versus AD, P<0.01). Furthermore, VEGF protein expression was lower in the AD group than that in the MCI group (P<0.01) (Fig. 1B and C). These results indicate that the mRNA and protein expression levels of VEGF in the CSF are decreased in patients with MCI and AD.

### Expression levels of VEGF mRNA and protein in the serum are decreased in patients with MCI and AD

**Figure 2:**
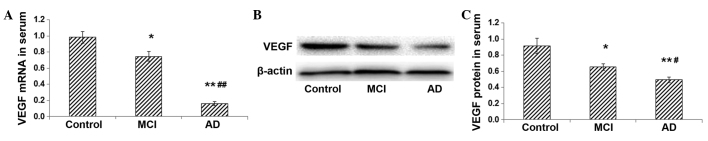
Changes in the expression levels of VEGF mRNA and protein in the serum of patients with dementia. (A) mRNA expression of VEGF in the serum of patients with dementia was measured by the reverse transcription-quantitative polymerase chain reaction. (B) VEGF protein expression in the serum of patients with dementia was detected through western blot analysis. (C) Statistical analysis of VEGF protein expression in the serum. Compared with the control group, ^*^P<0.05 and ^**^P<0.01; compared with the MCI group, ^#^P<0.05 and ^##^P<0.01. MCI, mild cognitive impairment; AD, Alzheimer’s disease; VEGF, vascular endothelial growth factor.

To further confirm the association between VEGF expression and the pathogenesis of AD, the serum expression of VEGF was determined. The RT-qPCR results showed that the serum expression level of VEGF mRNA was significantly lower in the MCI group than that in the control group (P<0.05) (Fig. 2A). In the AD group, the serum VEGF mRNA expression was further decreased and was significantly different from that in the MCI group (P<0.01) (Fig. 2A). Western blot analysis demonstrated that the protein expression level of VEGF in the serum was markedly lower in the MCI group than that in the control group (P<0.05), and it was further decreased in the AD group (P<0.01) (Fig. 2B and C). These results indicate that the VEGF mRNA and protein expression levels in the serum decrease with the increase in disease severity.

### Expression levels of miRNA-210 are reduced in the CSF and serum of patients with MCI and AD

**Figure 3:**
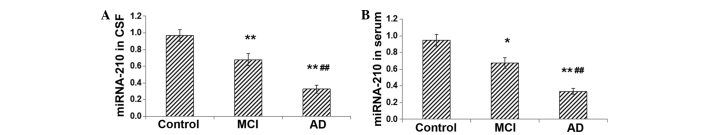
Changes in the expression levels of miRNA-210 in the CSF and serum of patients with dementia. miRNA-210 expression in the (A) CSF and (B) serum was detected by the reverse transcription-quantitative polymerase chain reaction. Compared with the control group, ^*^P<0.05 and ^**^P<0.01; compared with the MCI group, ^##^P<0.01. MCI, mild cognitive impairment; AD, Alzheimer’s disease; miRNA-210, microRNA-210; CSF, cerebrospinal fluid.

Since VEGF is one of the targets of miRNA-210, the current study investigated whether the level of miRNA-210 was altered in patients with MCI and AD. The expression levels of miRNA-210 in the CSF and serum were determined using RT-qPCR. The results indicated that the levels of miRNA-210 in the CSF and serum were significantly decreased in the MCI group compared with those in the control group (for CSF, P<0.01; for serum, P<0.05) (Fig. 3A and B). Furthermore, in the AD group, the expression levels of miRNA-210 in the CSF and serum were markedly lower than those in the MCI group (both P<0.01) (Fig. 3A and B). These results indicate that, similar to VEGF, the expression levels of miRNA-210 in the CSF and serum decrease as the severity of AD increases.

## Discussion

AD is one of the most common types of dementia and is caused by chronic pathological changes in the central nervous system. The clinical features of AD include amnesia, spatial learning and memory disability, language barriers, apraxia, cognitive impairment, executive dysfunction, and changes in personality and behavior. The risk of the disease increases with age, with a higher incidence in individuals >70 years old. The onset and pathogenesis of AD remains incompletely understood (9). Under current medical standards, clinical diagnostic tools for AD include neuropsychological tests, hematological examination, neuroimaging, electroencephalography and CSF detection (10). Hematological examination is primarily used to detect concomitant diseases, complications and potential risk factors, and to help exclude dementia caused by other medical conditions. Although it has good sensitivity and specificity, the application of CSF detection is currently limited due to the lack of uniform testing and sample processing methods, which could lead to clinical diagnostic errors and missed diagnoses. For these reasons, it is necessary to determine other more reliable and stable genetic markers in the CSF and blood detection.

Cerebral vascular changes, which contribute to the pathological changes in nerve cells, may be one of the important risk factors for a number of neurological diseases. These pathological changes result in cerebral insufficiency, low brain oxygen tension, low oxygen exchange rate and CO_2_ poisoning (11). It has been found that the temporal parietal and prefrontal lobes in the brains of patients with AD exhibit significant atrophy and that there are numerous neurofibrillary tangles in the nerve cells; these observations are associated with microvascular pathological changes (12). Brain vascular lesions may also cause ischemia and hypoxia, which induce β-amyloid deposition, abnormal phosphorylation of tau protein and neuronal degeneration and death (13). These findings indicate that there are vascular pathological changes in the brains of individuals with AD, which may contribute to the occurrence and development of the disease. In the present study, certain patients with AD were subjected to intracranial pressure monitoring, brain oxygen tension monitoring and computed tomography, and the results showed increased intracranial pressure, decreased oxygen partial pressure and intracranial vascular blockage and/or stenosis, which indirectly demonstrated the presence of vascular lesions in the brains of the patients with AD (data not shown).

VEGF is one of the most effective angiogenic growth factors in the human body and is able to promote angiogenesis and increase the blood supply (3). Solerte *et al* (14) found that the level of immune cell-released VEGF in patients with AD was lower than the normal level. A decrease in the immune cell-released VEGF could reduce the protection of neuronal cells and inhibit microvascular nutritional factors, resulting in an increase in brain injuries under hypoxia (14). Detection of the level of VEGF in the brain tissue could indicate the neurodegenerative pathological processes in cases of AD; however, considerable difficulty exists in the detection of VEGF expression in biopsy brain tissue samples. Furthermore, it has been demonstrated that, when brain lesions are severe, VEGF can enter the blood circulation from the brain due to the rupture of nerve cells (15). The detection of VEGF levels in the CSF can therefore be used as an indicator for VEGF expression in the brain. In the present study, the mRNA and protein expression levels of VEGF in the CSF and serum were significantly downregulated in the MCI group when compared with those in the control group. Furthermore, the expression of VEGF was further deceased in the AD group relative to that in the MCI group. These results indicate a downward trend in VEGF expression with increasing severity of dementia. The expression level of VEGF was decreased in both the CSF and serum; however, the expression levels differed slightly between the two, indicating the differential sensitivities of the same indicator in the CSF and serum in reflecting the pathological conditions of AD.

There are a number of factors affecting the regulation of mRNA expression. Recent studies have revealed that miRNAs are a class of endogenous, small, non-coding RNA molecules in cells that are important modulators for normal development and physiological and pathological conditions (16,17). miRNAs regulate target mRNAs in a negative feedback loop, cutting the mRNAs and inhibiting their translation. In the processes of tumor formation and development (18), angiogenesis and nerve repair (19), capillary formation (20) and ligament repair (21), miRNA-210 has been observed to upregulate VEGF to promote the growth of vascular cells. The present results demonstrated that miRNA-210 was downregulated with decreasing VEGF expression in the CSF and serum in patients with AD, which was in line with the results of other studies (18–21).

The results of the current study demonstrated that disease severity, VEGF expression and miRNA-210 level are associated with disease development. The expression of miRNA-210 in the CSF and serum may indirectly reflect the severity of dementia due to AD, and the regulation of miRNA-210 expression may affect the disease processes. The patients enrolled in the present study were categorized with the NINCDS-ADRDA diagnostic criteria amendment published by the NIA-AA, and the basic information about the patients, including their gender, age and medical history, was matched as far as possible. Despite this, the results may be limited due to the limited sample size and the regional differences among the patients, as well as the fact that there are numerous other factors associated with AD pathogenesis besides VEGF and miRNA-210 (20–24). Furthermore, the actions of miRNA-210 may differ from case to case, and so further studies are required to detail and validate the specific underlying mechanisms of miRNA-210 in cellular experiments, animal model studies and clinical trials.

In conclusion, the present results showed that the expression levels of VEGF mRNA and protein were significantly decreased in the CSF and serum of patients with MCI and AD, with an evident decreasing trend with increased disease severity. Notably, as a modulator of VEGF expression, the level of miRNA-210 also declined in the CSF and serum in patients with MCI and AD. These results demonstrate that miRNA-210 is not only indicative of AD pathogenesis, but may also provide novel insights into the prevention and treatment of the disease.

## Figures and Tables

**Table I:** Primer sets used for the reverse transcription-quantitative polymerase chain reaction.

Primer sets	Sequences
VEGF	
Forward	5′-TTGCCTTGCTGCTCTACCTC-3′
Reverse	5′-AAATGCTTTCTCCGCTCTGA-3′
β-actin	
Forward	5′-TGACGTGGACATCCGCAAAG-3′
Reverse	5′-CTGGAAGGTGGACAGCGAGG-3′
miRNA-210	
Forward	5′-CTGTGCGTGTGACAGCGGCTGA-3′
Reverse	5′-GCGAGCACAGAATTAATACGAC-3′
U6	
Forward	5′-CGCTTCGGCAGCACATATACTA-3′
Reverse	5′-CGCTTCACGAATTTGCGTGTCA-3′

VEGF, vascular endothelial growth factor; miRNA, microRNA.
